# The dihydropyridine calcium channel blocker benidipine prevents lysophosphatidylcholine-induced endothelial dysfunction in rat aorta

**DOI:** 10.1186/1423-0127-16-57

**Published:** 2009-06-26

**Authors:** Makoto Takayama, Kozo Yao, Michihito Wada

**Affiliations:** 1 Pharmacological Research Laboratories, Pharmaceutical Research Center, Kyowa Hakko Kirin Co, Ltd, 1188 Shimotogari, Nagaizumi-cho, Sunto-gun, Shizuoka-ken, 411-8731, Japan

## Abstract

**Background:**

Lysophosphatidylcholine (LPC), an atherogenic component of oxidized low-density lipoprotein, has been shown to induce the attenuation of endothelium-dependent vascular relaxation. Although benidipine, a dihydropyridine-calcium channel blocker, is known to have endothelial protective effects, the effects of benidipine on LPC-induced endothelial dysfunction remain unknown. We examined the effects of benidipine on the impairment of endothelium-dependent relaxation induced by LPC.

**Methods:**

Benidipine was administered orally to rats and aortas were then isolated. Aortic rings were treated with LPC and endothelial functions were then evaluated. Additionally, the effects of benidipine on intracellular calcium concentration ([Ca^2+^]_i_) and membrane fluidity altered by LPC in primary cultured rat aortic endothelial cells were examined. [Ca^2+^]_i _was measured using the fluorescent calcium indicator fura-2. Membrane fluidity was monitored by measuring fluorescence recovery after photobleaching.

**Results:**

Treatment with LPC impaired endothelial function. Benidipine prevents the impairment of relaxation induced by LPC. Acetylcholine elicited an increase in [Ca^2+^]_i _in fura-2 loaded endothelial cells. The increase in [Ca^2+^]_i _was suppressed after exposure to LPC. Plasma membrane fluidity increased following incubation with LPC. Benidipine inhibited the LPC-induced increase in membrane fluidity and impairment of increase in [Ca^2+^]_i_.

**Conclusion:**

These results suggest that benidipine inhibited LPC-induced endothelial dysfunction by maintaining increase in [Ca^2+^]_i_. Benidipine possesses membrane stabilization properties in LPC-treated endothelial cells. It is speculated that the preservation of membrane fluidity by benidipine may play a role in the retainment of calcium mobilization. The present findings may provide new insights into the endothelial protective effects of benidipine.

## Background

One of the pathological manifestations in atherosclerosis is the dysfunction of vascular endothelial cells [1]. Oxidized low-density lipoprotein (ox-LDL) is known to accumulate in atherosclerotic arterial walls [2]. A major bioactive ingredient of ox-LDL appears to be lysophosphatidylcholine (LPC), as this lysolipid can inhibit endothelium-dependent relaxation (EDR) [3,4]. One mechanism by which LPC causes impairment of EDR is to inhibit the release of nitric oxide (NO), which is dependent upon the intracellular calcium concentration ([Ca^2+^]_i_) [3,4]. The mechanism by which LPC interacts with endothelial cells to facilitate the inhibition of EDR remains unclear. LPC could inhibit receptor-mediated increases in [Ca^2+^]_i _in human umbilical vein endothelial cells by direct activation of protein kinase C (PKC) [5]. Activated PKC has been shown to inhibit receptor coupled-IP_3 _formation and subsequent increases in [Ca^2+^]_i _in response to agonists in endothelial cells [5]. On the other hand, it has been suggested that LPC induces membrane perturbation accompanied with receptor-G protein uncoupling in porcine aortic endothelial cells [6]. LPC has been shown to increase the fluidity of endothelial cell membranes and can be cytotoxic to endothelial cells [7,8]. It is possible that increased incorporation of LPC into the plasma membrane of endothelial cells may induce disruption of the receptor signal transduction system, thereby leading to impaired production of NO. These data suggest that LPC-induced changes may vary depending on the origin and culture of endothelial cells.

Benidipine hydrochloride (benidipine), a dihydropyridine-calcium channel blocker, has potent and long-acting antihypertensive effects [9]. We previously showed that benidipine has pharmacological properties which improve endothelial functions in hypertensive or hypercholesterolemic experimental models [10,11]. In cultured endothelial cells, benidipine inhibits LPC-induced vascular cell adhesion molecule-1 (VCAM-1) expression, reactive oxygen species (ROS) production and apoptosis [12-14]. Endothelial cells do not express L-type voltage-dependent calcium channels, which are the primary targets of dihydropyridine derivatives [15]. It has been suggested that the effects of benidipine are, in part, due to an anti-oxidant action or upregulation of endothelial nitric oxide synthase (eNOS) expression [12-14]. However, whether benidipine affects the LPC-induced dysfunction of vascular EDR remains unclear. In the present experiments, the effects of benidipine on the LPC-induced decrease in EDR in rat aortas were investigated and compared with that of other dihydropyridines. Additionally, the effects of benidipine on agonist-induced increases in [Ca^2+^]_i _attenuated by LPC were examined. Finally, the inhibitory potency of benidipine on LPC-induced membrane perturbation was assessed.

## Methods

### Animals

Male SD rats 7–8 weeks (Japan SLC Inc., Shizuoka, Japan) were used. All animals were kept at 19–25°C in a 12 hr light/dark cycle. Food and water were available ad libitum to all animals. This study was conducted in accordance with the Standards for Proper Conduct of Animal Experiments of Kyowa Hakko Kirin.

### Drugs

Benidipine and amlodipine besilate (amlodipine) were produced by Kyowa Hakko Kirin. Nifedipine, L-α-lysophosphatidylcholine (C16:0, LPC), L-phenylephrine hydrochloride (PE), acetylcholine chloride (ACh), pluronic F-127, phorbol 12-myristate 13-acetate (PMA), calphostin C and pyrrolidine dithiocarbamate (PDTC) were purchased from Sigma-Aldrich (St. Louis, MO, USA). Fura-2 acetoxy methylester was purchased from Wako Pure Chemical Industries (Osaka, Japan). Ro-31-8220 and phorbol 12, 13-dibutyrate (PDB) were purchased from Nacalai Tesque (Kyoto, Japan). Butylated hydroxytoluene (BHT) was purchased from LKT Laboratories (St. Paul, MN, USA). 1-Acyl-2-[6-[N-(7-nitro-2-1,3-benzoxadiazol-4-yl)amino]hexanoyl]-sn-glycero-3-phosphocholine (NBD-PC) was purchased from Avanti Polar Lipids (Alabaster, AL, USA). Ascorbic acid was purchased from Kanto Kagaku (Tokyo, Japan). Benidipine, amlodipine and nifedipine were suspended in 0.5% w/v methylcellulose 400 cP (Wako Pure Chemical Industries) and administered orally. For the *in vitro *assay, benidipine, fura-2 acetoxy methylester, PMA, PDB, Ro-31-8220, calphostin C, PDTC, ascorbic acid and BHT were dissolved in dimethylsulfoxide (DMSO). LPC was dissolved in ethanol. Other chemicals were dissolved in distilled water.

### Vascular reactivity following oral administration of dihydropyridines

Rats were administered orally with benidipine (4 mg/kg), amlodipine (4 mg/kg), nifedipine (10 mg/kg) or vehicle in a volume of 5 mL/kg. The doses of benidipine and amlodipine used were those employed in a previous study and found to lower blood pressure in Dahl salt-sensitive hypertensive rats [16]. The dose of nifedipine used was selected on the basis of a study by Kubo et al [17]. The depressor effects of these drugs generally exhibit a steady state during 60–120 min following administration in rats [17-19]. Therefore, thoracic aortas were isolated under sodium pentobarbital anesthesia at 90 min following administration. Aortas were immediately placed in gassed (95%O_2_+5%CO_2_) Krebs-Henseleit solution comprising 119 mmol/L NaCl, 4.7 mmol/L KCl, 1.2 mmol/L MgSO_4_, 1.8 mmol/L CaCl_2_, 1.2 mmol/L KH_2_PO_4_, 24.9 mmol/L NaHCO_3 _and 11.1 mmol/L glucose, and then cut into 2–3 mm length rings. Aortic rings were attached to holders and then placed in an organ bath filled with 20 mL Krebs-Henseleit solution. Following 60 min equilibration, aortic rings were incubated with 5 μmol/L LPC or 0.05% ethanol for 60 min. Tissues were washed three times, and vasorelaxant responses were measured as previously described [10]. Rings were contracted to submaximal tone with 1 μmol/L PE and subsequently relaxed using the endothelium-dependent vasodilator ACh (0.001–1 μmol/L). The percentage was calculated by considering contractions obtained immediately prior to the addition of ACh as 100%.

### Culture of rat aortic endothelial cells

Primary cell cultures of endothelial cells were established using cells removed from thoracic aortas. Isolation of aortic endothelial cells was performed by scraping following exposure to 1 mg/mL collagenase (Wako Pure Chemical Industries) for 60 min. Cells were seeded onto 35 mm diameter glass base dishes (12 mm diameter glass, Asahi Techno Glass, Chiba, Japan) in HuMedia-EG2 (Kurabo, Osaka, Japan) growth medium containing of 2% (v/v) fetal bovine serum, 10 ng/mL human epidermal growth factor, 10 μg/mL heparin, 1 μg/mL hydrocortisone, 50 μg/mL gentamycin and 50 ng/mL amphotericin B according to the manufacturer's instructions. Cells were cultured at 37°C in humidified atmosphere of 95% air and 5% CO_2_, and grown subconfluently 4–7 days after isolation.

### Intracellular calcium measurement

It has been reported that LPC inhibits ACh-mediated vasodilation via inhibition of increases in [Ca^2+^]_i _in endothelial cells [3,4]. To investigate the effects of benidipine on LPC-attenuated [Ca^2+^]_i_responses to ACh, we measured [Ca^2+^]_i _in rat aortic endothelial cells. [Ca^2+^]_i _was monitored by measuring the fluorescence (F340/F380) of fura-2. Cells were treated with 5 μmol/L fura-2 acetoxy methylester and 0.04% pluronic F-127 in Hanks solution (Nissui Pharmaceutical, Tokyo) for 15 min at 37°C. Cells were washed three times and fluorescence signals were monitored using an Argus-50 fluorescence spectrophotometer (Hamamatsu photonics, Shizuoka, Japan) at room temperature. Response to 3 μmol/L ACh was obtained and was then followed by washing. Cells were incubated with 3 μmol/L LPC or 0.03% ethanol for 30 min followed by washing. After washing, cells were treated with 3 μmol/L ACh. Changes in responses to ACh were expressed as percent values. Benidipine (1 or 10 nmol/L) or 0.1% DMSO was simultaneously added with LPC or ethanol for 30 min to evaluate the effects on ACh-induced increases in [Ca^2+^]_i_. The PKC activators PMA and PDB, and PKC inhibitors Ro-31-8220 and calphostin C were also examined in an effort to delineate the involvement of PKC upregulation in LPC-induced endothelial dysfunction. It has been reported that anti-oxidants can mimic the action of benidipine as manifested by the inhibition of LPC-induced VCAM-1 expression and ROS production [12,13]. Therefore, we included the anti-oxidants ascorbic acid, PDTC and BHT with LPC to examine the anti-oxidant effects on LPC-induced endothelial dysfunction.

### Membrane fluidity measurement

To evaluate membrane fluidity, fluorescence recovery after photobleaching (FRAP) experiments were performed. Cells were treated with 2 μg/L NBD-PC in Hanks solution for 15 min at 37°C. Cells were washed three times, and then incubated in the presence of 3 μmol/L LPC or ethanol for 30 min. Benidipine (10 nmol/L) or DMSO was added with LPC or ethanol. After three washes, FRAP experiments utilizing LSM510META (Carl Zeiss MicroImaging GmbH, Göttingen, Germany) were performed at room temperature. The fluorescence of a small area of the labeled cell was photobleached using a laser beam (excitation: 458 nm 10%, bleaching: 458, 488, 514, 543, 633 nm 100%, iteration: 800). After photobleaching, the fluorescent intensity in this bleached area was recovered by redistribution of the dye. The recovery percent was calculated one second following the end of the photobleaching.

### Data analysis

All values are expressed as mean ± standard error (S.E.). Statistical analyses were performed using SAS (SAS Institute, Inc., Cary, NC). For the comparison between 2 groups, an F-test was employed to evaluate a difference on variances. The Student's *t*-test was performed when significance on variance was not observed. The Aspin-Welch test was performed when significance on variances was observed. Differences in multigroup were assessed using 1-way ANOVA and the Dunnett test after confirmation of significance on variances was not observed by the Bartlett test. Differences were considered statistically significant at P < 0.05.

## Results

### Inhibitory effects of benidipine on LPC-induced attenuation of EDR in rat aortas

**Figure 1:**
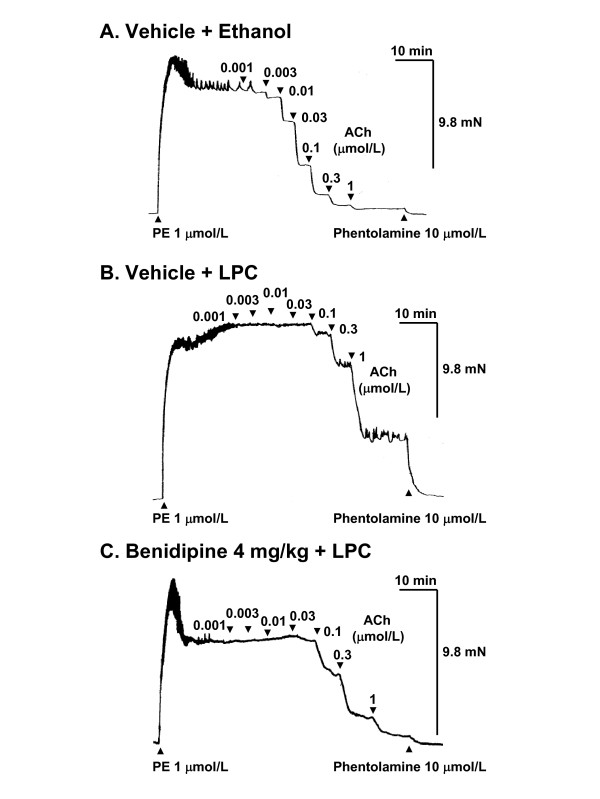
**Tracings showing relaxant response to ACh (endothelium-dependent vasodilator) in isolated rat aorta**. Benidipine or vehicle was administered orally to rats. Thoracic aortas were isolated at 90 min following benidipine administration. Aortas were cut into rings, and incubated with LPC (5 μmol/L) or ethanol (0.05%) for 60 min. After washing, the rings were pre-contracted using PE (1 μmol/L) and subsequently relaxed using ACh. ACh evoked relaxation in the aortic ring isolated from vehicle-treated rat (A). EDR was markedly attenuated following incubation with LPC (B). LPC-induced attenuation of EDR was inhibited in benidipine-treated rat (C).

**Figure 2:**
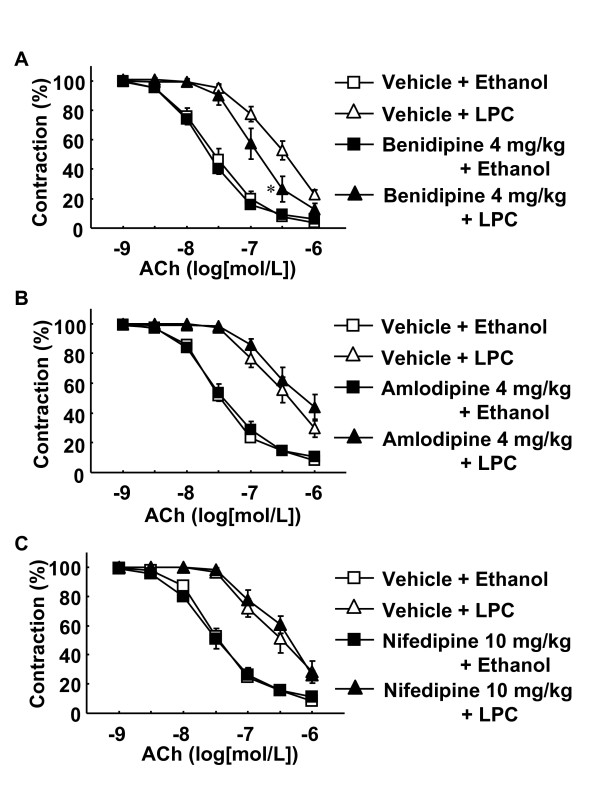
**Effects of benidipine (A), amlodipine (B) and nifedipine (C) on LPC-induced attenuation of endothelium-dependent relaxation**. Drugs were administered orally to rats. Rats were treated as described for Figure 1. Data are expressed as percentage of PE-induced contraction. Each value represents the mean ± S.E. of 6–10 experiments. *P < 0.05 vs. vehicle + LPC group.

PE induced contraction and ACh elicited relaxation in isolated muscle strips from rat thoracic aortas (Figure 1A). ACh-induced relaxation was markedly impaired following LPC challenge (Figure 1B, 2A, B and 2C). Orally administered benidipine inhibited LPC-induced attenuation of EDR (Figure 1C and 2A). In contrast, amlodipine and nifedipine did not affect LPC-induced attenuation of EDR (Figure 2B and 2C). Benidipine, amlodipine and nifedipine did not significantly inhibit PE-induced contraction (data not shown).

### Reversal effects of benidipine on LPC-induced suppression of [Ca^2+^]_i _increase in endothelial cells

**Figure 3:**
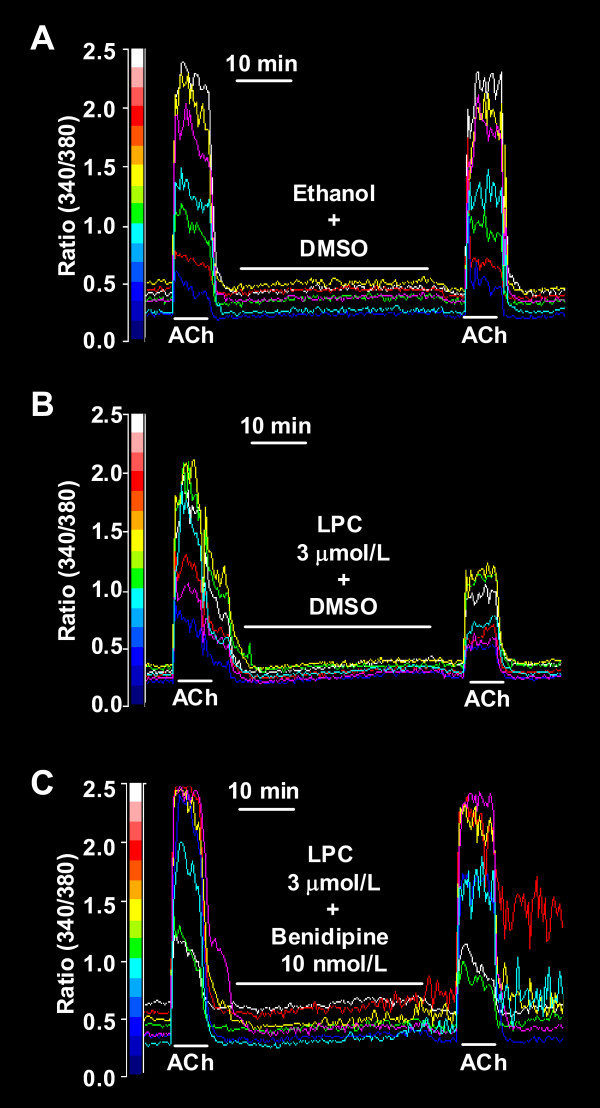
**Tracings showing [Ca^2+^]_i _(indicated by fluorescence ratio of fura-2 at 340 nm and 380 nm) transient in rat aortic endothelial cells**. ACh (3 μmol/L) elicited increases in [Ca^2+^]_i _in fura-2-loaded endothelial cells. Vehicles of LPC and benidipine had no effect on ACh-induced increases in [Ca^2+^]_i _(A). LPC (3 μmol/L) decreased ACh-induced increases in [Ca^2+^]_i _(B). Benidipine (10 nmol/L) prevented LPC-induced suppression of increases in [Ca^2+^]_i _(C).

**Figure 4:**
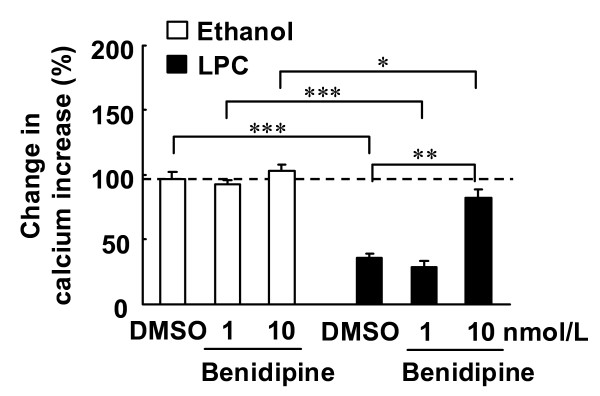
**Effects of benidipine with or without LPC on ACh-induced increases in [Ca^2+^]_i_**. Endothelial cells were treated as described for Figure 3. Benidipine (1 or 10 nmol/L) or DMSO was simultaneously added with LPC or ethanol. Changes in ACh-induced increases in calcium are expressed as percentage fluorescence value of ACh-induced increases in calcium prior to LPC or ethanol treatment. Each value represents the mean ± S.E. of 7 cells. *P < 0.05, **P < 0.01, ***P < 0.001 compared between indicated groups.

**Figure 5:**
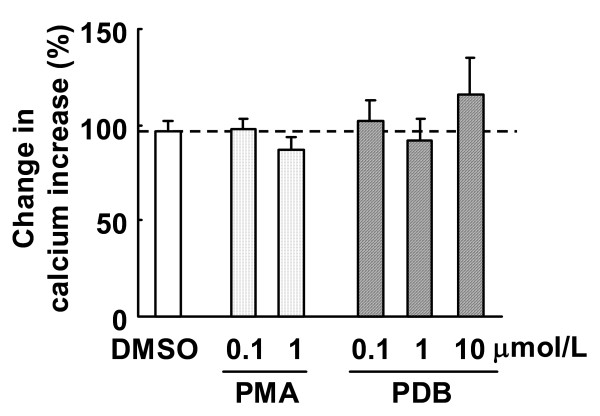
**Effects of PKC activators on ACh-induced increases in [Ca^2+^]_i_**. Fura-2 loaded endothelial cells were treated with ACh (3 μmol/L) followed by washing. Cells were then incubated with PMA, PDB or DMSO for 30 min. After washing, ACh (3 μmol/L) was added once again. Changes in ACh-induced increases in calcium are expressed as percentage fluorescence value of ACh-induced increases in calcium prior to LPC or ethanol treatment. Each value represents the mean ± S.E. of 7 cells.

**Figure 6:**
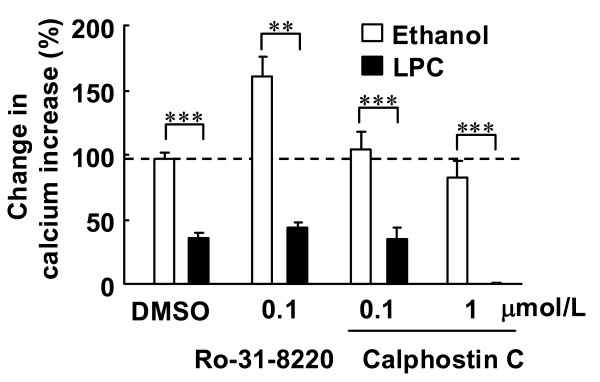
**Effects of PKC inhibitors with or without LPC on ACh-induced increases in [Ca^2+^]_i_**. Fura-2 loaded endothelial cells were treated with the protein kinase C inhibitors Ro-31-8220 and calphostin C for 30 min. ACh (3 μmol/L) was added before and after treatment. Data are expressed as percentage value of ACh-induced increases in calcium prior to treatment. Each value represents the mean ± S.E. of 7 cells. **P < 0.01, ***P < 0.001 compared between indicated groups.

**Figure 7:**
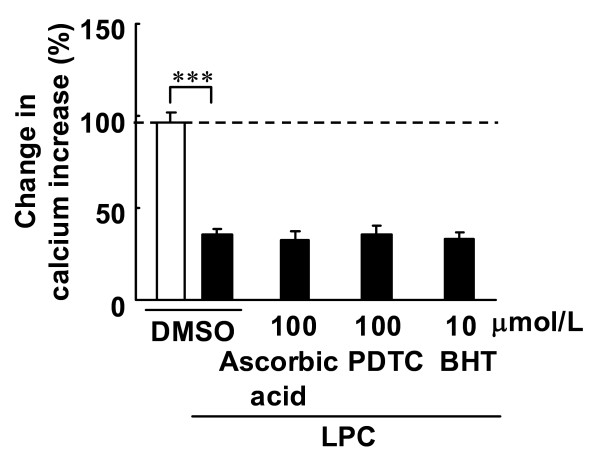
**Effects of anti-oxidants on ACh-induced increases in [Ca^2+^]_i _with LPC**. Fura-2 loaded endothelial cells were treated with the anti-oxidants ascorbic acid, PDTC and BHT for 30 min. ACh (3 μmol/L) was added before and after treatment. Data are expressed as percentage value of ACh-induced increases in calcium prior to the treatment. Each value represents the mean ± S.E. of 7 cells. ***, P < 0.001 compared between indicated groups.

In fura-2 loaded endothelial cells, ACh induced an increase in [Ca^2+^]_i _(Figure 3A). The increase in [Ca^2+^]_i _was inhibited by 3 μmol/L LPC (Figure 3B and 4), and the simultaneous addition of 10 nmol/L benidipine prevented LPC-induced suppression of increases in [Ca^2+^]_i _(Figure 3C and 4). In the absence of LPC, benidipine had no effect on ACh-induced increases in [Ca^2+^]_i _at 1 and 10 nmol/L (Figure 4). The PKC activators PMA and PDB did not alter ACh-induced increases in [Ca^2+^]_i _(Figure 5). The PKC inhibitors Ro-31-8220 and calphostin C did not affect the LPC-induced suppression of increases in [Ca^2+^]_i _(Figure 6). The antioxidants ascorbic acid, PDTC and BHT also did not affect the LPC-induced suppression of increases in [Ca^2+^]_i _(Figure 7).

### Effects of LPC and benidipine on membrane fluidity

**Figure 8:**
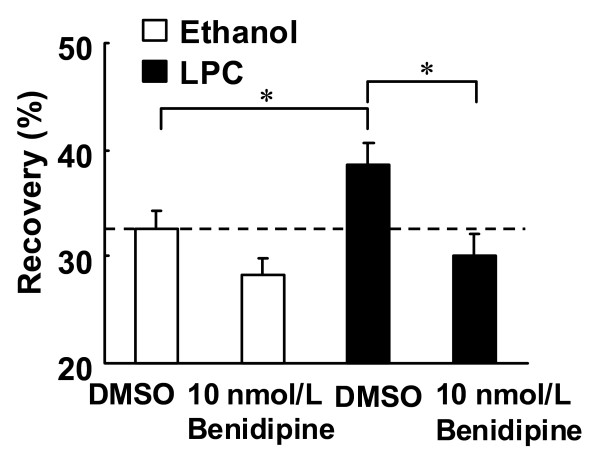
**Effects of benidipine on fluorescence recovery one second after the end of photobleaching**. NBD-PC loaded endothelial cells were treated with LPC (3 μmol/L) or ethanol (0.03%) for 30 min. Benidipine (10 nmol/L) or DMSO was simultaneously added with LPC or ethanol. Each value represents the mean ± S.E. (n = 18–28). *P < 0.05 compared between indicated groups.

The movement of dye in the FRAP experiment indicated that LPC increased the membrane fluidity of endothelial cells. Benidipine at 10 nmol/L normalized membrane fluidity in the presence of LPC (Figure 8).

## Discussion

In the present study, we demonstrated that LPC inhibited receptor-mediated EDR and increases in [Ca^2+^]_i _in endothelial cells. Reduced production of NO in LPC-treated aorta likely results from inhibition of ACh-induced increases in [Ca^2+^]_i_. Benidipine treatment prevented LPC-induced attenuation of EDR. In endothelial cells, benidipine prevented LPC-induced attenuation of increases in [Ca^2+^]_i_. These results suggest that benidipine inhibits LPC-induced impairment of EDR by retainment of [Ca^2+^]_i _mobilization in endothelial cells. The oxidative stress caused by LPC is postulated to be responsible for the inhibition of EDR [20]. Endothelial dysfunction is associated with increased inactivation of NO by the superoxide anion (O^2-^) resulting in the formation of peroxynitrite (ONOO^-^) [21]. It is known that some dihydropyridine derivatives can augment NO release from endothelial cells probably through anti-oxidation [22]. It has been suggested that nifedipine increases the bioavailability of endothelial NO by reducing the production of ROS [23]. Benidipine is also known to possess antioxidant activity [24]. Therefore inhibition of ROS production by benidipine may result in increased bioavailability of NO. If the anti-oxidative properties of benidipine account for the inhibition of LPC-induced endothelial dysfunction, anti-oxidants should restore EDR. Previous reports have shown that ascorbic acid and PDTC can mimic the action of benidipine as manifested by the inhibition of LPC-induced VCAM-1 expression and ROS production [12,13]. BHT is a lipid-soluble anti-oxidant which provides protection against experimental atherosclerosis [25]. In the present study, those antioxidants did not alter the LPC-induced inhibition of increases in [Ca^2+^]_i_. The lack of effects of the antioxidants suggested that the effects of benidipine did not emanate from its anti-oxidant activity.

The precise mechanisms responsible for the inhibitory effect of LPC on EDR have not been elucidated [3-8]. There is some evidence to suggest that LPC could cause receptor-G-protein uncoupling [6]. In intact rabbit aorta, LPC induced attenuation of ACh-mediated increases in [Ca^2+^]_i _[3]. The inhibitory effect of LPC may result from direct interaction with the plasma membrane of endothelial cells [7,8]. An increase in LPC may alter physiological properties of the plasma membrane such as membrane fluidity and permeability [7,8]. This alteration in membrane fluidity may also displace boundary lipids around integral protein effector systems, which might in turn interfere with protein structure and enzymatic activities [26]. On the other hand, it has been reported that PKC activation by LPC inhibited thrombin-mediated increases in [Ca^2+^]_i _in human umbilical vein endothelial cells [5]. In our experiment, the PKC inhibitors Ro-31-8220 and calphostin C did not inhibit LPC-induced [Ca^2+^]_i _suppression. The direct PKC activators PMA and PDB had no effect on ACh-induced increases in [Ca^2+^]_i_. These findings suggest that PKC activation does not account for the inhibition of ACh-induced increases in [Ca^2+^]_i _in rat aortic endothelial cells. We showed that LPC induced significant increases in membrane fluidity. It was speculated that LPC probably induces the suppression of ACh-mediated increases in [Ca^2+^]_i _by increasing membrane fluidity. However, the mechanism underlying ACh-induced increases in [Ca^2+^]_i _remain unclear. Further studies are needed to rule out a causal relationship between membrane fluidity and increases in [Ca^2+^]_i_.

Benidipine inhibited the LPC-induced suppression of increases in [Ca^2+^]_i _at 10 nmol/L. LPC significantly increased relative membrane fluidity. In the presence of LPC, benidipine preserved an adequate level of membrane fluidity at 10 nmol/L. Benidipine possesses high liposolubility and affinity for cell membranes compared with amlodipine and nifedipine [9]. It seems likely that improved membrane fluidity with incorporated benidipine accounts for the inhibition by benidipine of LPC-induced suppression of increases in [Ca^2+^]_i_. In the present study, benidipine exclusively inhibited LPC-induced attenuation of EDR. It seems that the unique effects of benidipine are due to its high affinity for the cell membrane. It has been reported that the maximum plasma drug concentration of benidipine after oral administration to rats at a dose of 3 mg/kg, which is an antihypertensive dosage of spontaneously hypertensive rats, is 36 nmol/L (19.5 ng/mL) [18,27]. Therefore, the plasma concentration of benidipine is sufficient to preserve adequate membrane fluidity and associated functional changes in endothelial cells when administered in antihypertensive dosages.

It has been shown that benidipine inhibits LPC-induced expression of adhesion molecules, the production of ROS and apoptosis in endothelial cells [12-14]. The relationship between membrane fluidity and the mechanisms underlying these LPC-induced endothelial impairments remain poorly understood. In the present study, we did not perform the experiments to prove or disprove involvement of membrane perturbation in endothelial impairments. Therefore, the role of membrane stabilization in the inhibitory effects on LPC-induced endothelial impairment needs to be clarified in future studies.

LPC is associated with ox-LDL and is localized in atherosclerotic plaques in high concentrations [1,2]. The lysolipid may play an important atherogenic role during initial stages of the atherosclerotic process [28]. In endothelial cells, benidipine inhibits LPC-induced expression of adhesion molecules, production of ROS and apoptosis, in addition to impairment of vascular EDR [12-14]. These endothelial protective effects of benidipine may play an important role in the mechanism underlying protection against LPC-induced endothelial dysfunction associated with the early atherosclerotic process.

## Conclusion

Benidipine prevented LPC-induced attenuation of EDR in rat aorta. In endothelial cells, the LPC-induced decrease in ACh-mediated calcium mobilization and increase in membrane fluidity were inhibited by benidipine. From the present investigation, it is speculated that the inhibitory activity of benidipine against LPC-induced attenuation of EDR is related to its action involving membrane stabilization.

## Abbreviations

ACh: acetylcholine chloride; BHT: Butylated hydroxytoluene; [Ca^2+^]_i_: intracellular calcium concentration; EDR: endothelium-dependent relaxation; eNOS: endothelial nitric oxide synthase; DMSO: dimethylsulfoxide; LPC: L-α-lysophosphatidylcholine (C16:0); NBD-PC: 1-Acyl-2-[6-[N-(7-nitro-2-1,3-benzoxadiazol-4-yl)amino]hexanoyl]-sn-glycero-3-phosphocholine; NO: nitric oxide; ox-LDL: oxidized low-density lipoprotein; PDB: phorbol 12, 13-dibutyrate; PDTC: pyrrolidine dithiocarbamate; PE: L-phenylephrine hydrochloride; PMA: phorbol 12-myristate 13-acetate; ROS: reactive oxygen species; VCAM-1: vascular cell adhesion molecule-1.

## Competing interests

The authors declare that they have no competing interests.

## Authors' contributions

MT designed and performed experiments, and prepared a draft of the manuscript. KY and MW revised the drafted manuscript.
